# A chromosome-level genome assembly of the Asian arowana, *Scleropages formosus*

**DOI:** 10.1038/sdata.2016.105

**Published:** 2016-12-06

**Authors:** Jia Li, Chao Bian, Yinchang Hu, Xidong Mu, Xueyan Shen, Vydianathan Ravi, Inna S. Kuznetsova, Ying Sun, Xinxin You, Ying Qiu, Xinhui Zhang, Hui Yu, Yu Huang, Pao Xu, Ruobo Gu, Junmin Xu, László Orbán, Byrappa Venkatesh, Qiong Shi

**Affiliations:** 1 Shenzhen Key Lab of Marine Genomics, Guangdong Provincial Key Lab of Molecular Breeding in Marine Economic Animals, BGI, Shenzhen 518083, China; 2 Key Laboratory of Tropical & Subtropical Fishery Resource Application & Cultivation, Ministry of Agriculture, Pearl River Fisheries Research Institute, Chinese Academy of Fishery Sciences, Guangzhou 510380, China; 3 Reproductive Genomics Group, Temasek Life Sciences Laboratory, Singapore 117604, Singapore; 4 Institute of Molecular and Cell Biology, A*STAR, Biopolis, Singapore 138673, Singapore; 5 Laboratory of Chromosome Structure and Function, Department of Cytology and Histology, Biological Faculty, Saint Petersburg State University, Saint-Petersburg 198504, Russia; 6 Freshwater Fisheries Research Center, Chinese Academy of Fishery Sciences, Wuxi 214081, China; 7 BGI-Zhenjiang Institute of Hydrobiology, Zhenjiang 212000, China; 8 Marine Research Center, College of Life Sciences, Shenzhen University, Shenzhen 518060, China

**Keywords:** Genome, Bioinformatics, DNA sequencing, Ichthyology

## Abstract

Asian arowana (*Scleropages formosus*), an ancient teleost belonging to the Order Osteoglossomorpha, has been a valuable ornamental fish with some varieties. However, its biological studies and breeding germplasm have been remarkably limited by the lack of a reference genome. To solve these problems, here we report high-quality genome sequences of three common varieties of Asian arowana (the golden, red and green arowana). We firstly generated a chromosome-level genome assembly of the golden arowana, on basis of the genetic linkage map constructed with the restriction site-associated DNA sequencing (RAD-seq). In addition, we obtained draft genome assemblies of the red and green varieties. Finally, we annotated 22,016, 21,256 and 21,524 protein-coding genes in the genome assemblies of golden, red and green varieties respectively. Our data were deposited in publicly accessible repositories to promote biological research and molecular breeding of Asian arowana.

## Background & Summary

Asian arowana or Asian dragonfish (*Scleropages formosus*; Osteoglossidae, Osteoglossiformes, Osteoglossomorpha) represents an ancient lineage of teleost fish. The phylum Osteoglossomorpha is one of the basal branching lineages of teleosts with fossil records from the late Jurassic^1^. Because of its bright and shiny body colour, arowana has been considered as one of the most expensive ornamental fishes around the world^2^. At least five naturally occurring colour varieties of arowana have been identified. Among them, the red, golden and green varieties are the most popular. Within the three varieties, the red arowana has the highest commercial value, whilst the green one has the lowest price. In recent times the population size of arowana in its native habitat has declined due to overfishing. As a result, Asian arowana has been listed as an endangered species by the Convention on International Trade in Endangered Species of Wild Fauna and Flora (CITES) Appendix I since 1980 (ref. 3). It therefore raises people’s awareness about this endangered ornamental fish to some extent.

The Asian arowana, as a well-known representative of the Osteoglossiformes, possesses many primitive characters such as toothed tongue bone. On the other hand, arowana has evolved several derived traits such as mouth brooding and air-breathing function of the swim bladder. It is therefore considered as an important model for exploration of the teleost diversity. Furthermore, the genetic basis of body colour variations and the sex-determining mechanisms of arowana remain largely unknown.

Our previous study published in Scientific Reports^4^ has reported a primary genome assembly of a female golden arowana and the draft genomes of the green and red varieties. The genomic and transcriptome comparisons among these three varieties have interpreted the possible molecular mechanism of colour variations. In addition, a potential sex chromosome was identified, revealing a solid clue for the sex-determination of arowana.

To construct these genomes, we extracted genomic DNAs from the three varieties of arowana and subsequently sequenced up to 100-fold coverage using the Illumina HiSeq2000 platform. After filtering low quality reads, we applied the SOAPdenovo2.2 (ref. 5) to assemble the clean reads of the three varieties separately. We obtained scaffold N50 values of 5.96 Mb (golden arowana), 1.63 Mb (red arowana) and 1.85 Mb (green arowana) respectively. The assembled genome sizes are 779, 753 and 759 Mb respectively, which are consistent with the estimates by *k*-mer analyses (Table 1). By using *de novo*-assembled transcipts and the CEGMA method^6^, we confirmed good completeness and accuracy of the three assembled genomes.

**Figure 1:**
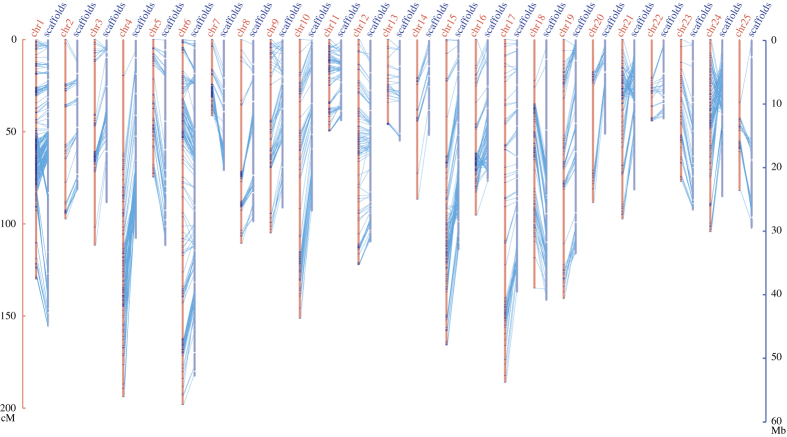
Construction of the 25 linkage groups (or pseudo-chromosomes) of golden arowana based on RAD-seq. Markers on the scaffolds (dark blue) were aligned and reordered onto the corresponding chromosomes (chr; in the orange color). The length for the axis is centimorgan (cM; for the chromosomes) or millionbase (Mb; for the scaffolds).

To construct chromosome-level assembly of the golden arowana, we performed restriction site-associated DNA sequencing (RAD-seq), on basis of 94 F2 individuals from red grad 1 and Malaysian golden crossback, to generate a high-density genetic linkage map. Ultimately, we identified a total of 22,881 single-nucleotide polymorphisms (SNPs), in which 5,740 SNPs were clustered into 25 linkage groups (Fig. 1). The genetic linkage map spanned a genetic distance of 3,240 cM. According to the refined SNPs and their corresponding scaffolds, we calculated the size of the chromosomes-level assembly up to 683 Mb, which accounts for 87.7% of the achieved genome assembly. The high-quality chromosome-level assembly of golden arowana, along with the high-density genetic linkage map, will provide a valuable resource for further comparative genomic studies.

Transposable elements (TEs) in the three assembled genomes were examined. We observed that TEs account for 27, 27 and 28% of the genome assemblies in golden, red and green varieties, respectively (Table 1). Multiple methods for gene annotation, including *ab initio*, transciptome and homology based gene prediction, were employed to generate refined gene sets, which contain 22,016 (golden arowana), 21,256 (red arowana) and 21,524 (green arowana) protein-coding genes, respectively.

The availability of a high-quality reference genome of the golden arowana provides a good opportunity for biological characterization and molecular breeding of arowana.

## Methods

These methods are expanded from detailed descriptions in our previous work^4^.

### Fish sample preparation

Experiments in China were approved by the Institutional Review Board on Bioethics and Biosafety of BGI and performed according to related guidelines. Animal experiments in Singapore were approved by Institutional Animal Care and Use Committee (approval ID: TLL(F)-10-003) of Temasek Life Sciences Laboratory and performed in accordance with its guidelines.

#### Fish samples for Illumina genome sequencing

Second filial generation (F2) individual samples of arowana, including one golden arowana (within 2 years old), one red arowana (within one-year-old) and one green arowana (within one-year-old), were obtained from Pearl River Fisheries Research Institute, Chinese Academy of Fishery Sciences, China.

#### Fish samples for RAD sequencing

The Qian Hu fish farm (Singapore) obtained F1 hybrid individuals that originated from crossing two unrelated and genetically divergent founder (F0) Asian arowana grandparents (Red grade 1×Malaysian golden varieties). Previously, we have generated two mapping families by crossing two pairs of these F1 hybrid brooders. The fostering methods of arowana are described in detail in our related work^4^. We used 94 F2 progenies and their parents for RAD sequencing to construct the high-density genetic map.

### Library construction, sequencing and genome assembly

#### Library construction and sequencing

High-quality genomic DNAs of three arowana varieties were extracted from the mixture of three tissues (muscle, skin and liver) independently using Puregene Tissue Core Kit A (Qiagen, MD, USA) for constructing libraries with different insert-sizes (170 bp to 40 kb; see more details in Table 2). Overall, we generated 16 pair-end libraries (8 for the golden arowana, 4 for the red arowana and 4 for the green variety, respectively) with the standard operating procedure provided by Illumina (San Diego, USA). Pair-end sequencing with the whole genome sequencing (WGS) strategy was performed on the Illumina HiSeq2000 platform using the standard operating procedure.

#### Raw data filtering

Raw reads were subjected to quality filtering (Raw Data Clean Procedure). Details for the disciplines of filtering were described in our related work^4^. The SOAPfilter v2.2 software (http://soap.genomics.org.cn/index.html) with the optimized parameters (-y -p -g 1 -o clean -M 2 -f 0) was chosen to remove low-quality bases and PCR-replicates as well as adaptor sequences. Finally, we obtained 74.07 Gb (golden arowana), 75.60 Gb (red arowana) and 60.40 Gb (green arowana) of clean reads.

#### *k*-mer analysis

A *k*-mer indicates a K-bp length nucleotide segment of sequencing reads. A raw sequencing read with a total length of L bp contains (L-K+1) *k*-mers^7^. Details of the *k-*mer analysis were provided in our related work^4^. Finally, we estimated that the genome sizes of the golden, red and green varieties are 822, 949 and 897 Mb, respectively.

#### Genome assembly

The filtered reads were assembled using SOAPdenovo2 v2.04.4 (ref. 5) software with optimized parameters (pregraph -K 25 -d 1; contig -M 1; scaff -F -b 1.5 -p16) to generate contigs and original scaffolds. The generated genome assemblies span approximately 779, 753 and 759 Mb for the golden, red and green varieties, respectively. Finally, the scaffold N50 values reached 5.96, 1.63 and 1.85 Mb for the golden, red and green varieties respectively (Table 1).

### RAD sequencing, genetic map construction, and chromosome-level assembly

#### RAD sequencing

High-quality genomic DNAs were extracted from the scales and/or fin clips of the 94 progeny individuals and their parents for RAD sequencing by using Mag Attract HMW DNA Kit (Qiagen, MD, USA). The DNAs were subsequently digested with the restriction endonuclease EcoRI and divided into 3 RAD libraries^8^. Sequencing was performed by an Illumina HisSeq2000 platform. Finally, 72.8 Gb of reads with 101-bp length (the average depth is 1×) were obtained.

#### RAD SNP calling

After performing the above-mentioned Raw Data Clean Procedure to filter the adaptor sequences and remove low-quality reads, we re-aligned the clean reads onto the golden assembly (reference) using SOAP2 v2.21 (ref. 9) software with optimized parameters (-m 100 -×888 -s 35 -l 32 -v 3 -p4). The SNPs were called using the Samtools^10^ in each individual. Those low-quality SNPs with the missing rates higher than 30% were discarded.

#### Genetic map construction

SNPs showing significant Mendelian segregation distortion (χ2 test, *P*<0.01, d.f.=1) were discarded. Then, the filtered SNPs were uploaded into JoinMap v4.1 (ref. 11) and analyzed with default parameters. The pairwise recombination estimations and logarithm of odds (LOD) scores of all SNPs were calculated, and SNPs pairs were then clustered into linkage groups at a LOD threshold of 10.0. Map distances (cM) were counted using the Regression mapping algorithm with the Kosambi function. All the SNP markers were clustered into 25 linkage groups (Fig. 1), which is consistent with our previously reported chromosome karyotype (2n=48 and one additional W chromosome)^12^.

#### Anchoring the genome assembly to the linkage groups

A total of 5,740 SNP markers which located on 194 scaffolds were used for chromosomal-level assembly. The relative order between anchored scaffolds on each chromosome was determined by the following disciplines. For those scaffolds with the number of SNPs more than two, the two SNPs with the best quality on each scaffold were chosen to determine the order and the orientation. However, the orientation of those scaffolds with only one SNP was not ordered due to the lack of enough markers. Subsequently these ordered scaffolds were directly anchored to the chromosomes. Finally, 87.65% of the assembled genome sequences were able to be anchored onto the 25 pairs of chromosomes (Fig. 1, Table 3).

### Analysis of repetitive sequences in the draft assembly

Firstly, a *de novo* repeat library was built using the RepeatModeller v1.04 (http://www.repeatmasker.org/RepeatModeler.html) and LTR_FINDER^13^ with default parameters. Subsequently, the RepeatMasker v3.2.9 (ref. 14) was used to align our sequences with the Repbase TE v14.04 (ref. 15) and the *de novo* repeat libraries to recognize transposable elements (TEs). The Tandem Repeat Finder v4.04 (ref. 16) with optimized parameters (Match=2, Mismatch=7, Delta=7, PM=80, PI=10, Minscore=50, and MaxPerid=2000) was performed to annotate tandem repeats. Furthermore, the RepeatProteinMask software v3.2.2 (http://www.repeatmasker.org) was conducted to identify TE relevant proteins in our generated assemblies. Finally, the TEs were estimated to account for 27.34, 27.93 and 28.04% of the golden, red and green arowanan genomes, respectively (Table 1).

### Genome annotation

In brief, we applied three independent methods to predict gene sets.

#### Homology annotation

We used the protein sequences from eight reported genomes (Table 4) to detect homology-based genes. All the protein sequences were downloaded from Ensembl (release 75). An all-against-all TblastN search was performed with an e-value of 10^−5^. Best hits in each of the analyzed genome were searched, and the potential gene structures were then predicted by using Genewise2.2.0 (ref. 17). Those genes with length less than 150 bp, or prematurely terminated or frame-shifted, were discarded.

#### *De novo* annotation

We implemented *de novo* similarity-based gene prediction using AUGUSTUS v.2.5 (ref. 18) with optimized parameters (pre-trained with 1,500 randomly selected genes from the homology annotation set). To avoid pseudogene annotation, the repetitive regions of three arowana varieties were masked as ‘N’ seqeunces. Then AUGUSTUS v2.5 and GENSCAN v1.0 (ref. 19) were performed on the three-draft repeat-mask genome sequences. Subsequently, three gene sets were filtered using the same method applied for the homology annotation.

#### RNA-seq annotation

RNAs were isolated independently from the skin tissues of three arowana varieties for RNA-seq. RNA-seq libraries were constructed using the Illumina mRNA-Seq Prep Kit (San Diego, CA, USA). Subsequent RNA sequencing was performed using the Illumina sequencing platform. Finally, 8.4 Gb of data were generated. After discarding the low quality reads, we used the Tophat1.2 (ref. 20) software to align filtered reads onto the corresponding genome sequences separately. Then the Cufflink^21^ software was employed to determine potential gene structures of the achieved alignments of Tophat.

#### Integration of annotation results

After merging all results generated from the above three methods, we applied GLEAN^22^ to obtain a comprehensive and non-redundant gene set. Expression levels of the GLEAN genes and the alignments of Tophat were calculated using the Cuffdiff package of Cufflink^21^ software with optimized parameters (-FDR 0.05 --geometric-norm TRUE -compatible-hits-norm TURE). To find the best hit of each deduced protein, we employed BlastP with an e-value of 10^−5^ to map the protein sequences of GLEAN results against the SwissPort and TrEMBL databases^23^ (Uniprot release 2011.06). The predicted protein sequences of the three arowana varieties were then aligned against the public databases (Pfam, PRINTS, ProDom and SMART) for detection of function motifs and domains. Ultimately, the GLEAN gene sets were filtered by the following three steps: 1) discarded genes with the length less than 150 bp, 2) discarded genes recognized as TEs, and 3) removed genes that were only obtained from the *de novo* method but without functional assignments and with an expression value lower than 1.

## Data Records

In our previous work^4^, annotated genome sequences of the golden, red and green arowana varieties were uploaded to GenBank under the GenBank assembly accession numbers GCA_001624265.1 (Data Citation 1), GCA_001624255.1 (Data Citation 2) and GCA_001624245.1 (Data Citation 3), respectively. The RAD-seq raw read files were stored at NCBI SRA under experiment accession numbers SRX1728941 to SRX1728946 (Data Citation 4). The RNA-seq raw read files can be found at NCBI SRA SRX1668426 to SRX1668432 (Data Citation 5). In this paper, the gene annotation information of three varieties of arowana are available from Dryad (Data Citation 6). The chromosome annotation of golden arowana are available from Dryad (Data Citation 6). Data are given in tabular, tab-delimited value format.

## Technical Validation

We took the following two steps to assess the generated genome assemblies. The first approach is Transcriptome evaluation. We used the Trinity^24^ software to *de novo* assemble the RNA sequences of skin tissues from three varieties, and then utilized the BLAT^25^ (E-value=10e^−6^, identity=90% and coverage>90%) to align the genome assemblies (Table 5). The second approach is CEGMA^6^ (Core Eukaryotic Genes Mapping Approach; http://korflab.ucdavis.edu/Datasets/genome_completeness, version 2.3) assessment with 248 conserved Core Eukaryotic Genes (CEGs) subjected to evaluation of the gene space completeness within the three assemblies. The evaluation results revealed a high coverage of more than 90% of gene coding-regions and over 95% of core eukaryotic genes, confirming the high-level completeness of the three genome assemblies (Table 6).

## Usage Notes

Except for the Joinmap v4.1 that was ran on a Windows system, the other softwares were run on a linux system. The optimized parameters are provided in the main text.

## Additional Information

**How to cite this article:** Li, J. *et al.* A chromosome-level genome assembly of the Asian arowana, *Scleropages formosus*. *Sci. Data* 3:160105 doi: 10.1038/sdata.2016.105 (2016).

**Publisher’s note:** Springer Nature remains neutral with regard to jurisdictional claims in published maps and institutional affiliations.

## Supplementary Material



## Figures and Tables

**Table 1:** Overview of genome assembly and annotation for the three varieties of Asian arowana.

**Colour variety**	**Golden**	**Red**	**Green**
Sequence coverage (×)	137.60	109.60	100.10
Estimated genome size (Mb)	828	949	897
Assembled genome size (Mb)	779	753	759
Scaffold N50 (Mb)	5.97	1.63	1.85
Contig N50 (kb)	30.73	60.19	62.80
Number of genes	22,016	21,256	21,524
Repeat content	27.34%	27.93%	28.04%

**Table 2:** Construction of the sequencing libraries.

**Species**	**Insert Size**	**Total Data (Gb)**	**Read Length**	**Sequence coverage (X)**
Golden arowana	170 bp	24.7	100	30.1
	500 bp	20.3	100	24.7
	800 bp	13.8	100	16.8
	2 kb	22.5	49	27.4
	5 kb	10.6	49	12.9
	10 kb	9.9	49	12.0
	20 kb	6.6	49	8.0
	40 kb	4.7	49	5.7
Total		113.1		137.6
Red arowana	250 bp	39.7	150	41.7
	500 bp	26.6	90	28.0
	2 kb	17.7	100	18.6
	5 kb	19.8	100	20.8
Total		103.8		109.6
Green arowana	250 bp	32.7	150	36.3
	500 bp	27.6	90	30.7
	2 kb	15.8	100	17.6
	5 kb	14.4	100	16.0
Total		90.5		100.1

**Table 3:** statistics of the anchored scaffolds for the golden variety.

**Colour variety**	**Golden**
No. of scaffolds (>2 kb)	554
N50 of Scaffold (Mb)	5.97
Assembled genome size (Mb)	779
No. of the anchored scaffolds	194
N50 of the anchored scaffolds (Mb)	7.26
Genome size of the anchored scaffolds (Mb)	683

**Table 4:** Versions of genome assemblies of the eight vertebrate species used for homology annotation.

**Common name**	**Species name**	**Version**
Human	*Homo sapiens*	Release 75
Zebrafish	*Danio rerio*	
Japanese fugu	*Takifugu rubripes*	
Spotted green pufferfish	*Tetraodon nigroviridis*	
Three-spined stickleback	*Gasterosteus aculeatus*	
Japanese medaka	*Oryzias latipes*	
Half-smooth tongue sole	*Cynoglossus semilaevis*	
Coelacanth	*Latimeria chalumnae*	

**Table 5:** Assessing the completeness of gene regions in the three genome assemblies by RNA-seq of skin tissue.

**Varieties of arowana**	**Reads Number**	**Total Length**	**Covered by assembly(%)**	**With >90% sequence in one scaffold**	**With >50% sequence in one scaffold**
Golden	312,697	419,386,592	95.10	89.85	96.74
Red	271,689	278,274,436	97.20	91.82	98.64
Green	329,917	427,288,534	96.07	91.45	98.41

**Table 6:** Coverage rates of core eukaryotic genes in the three assembled genomes by CEGMA.

**Varieties**	**Group 1**		**Group 2**		**Group 3**		**Group 4**	
	**Covered Number**	**Completeness (%)**	**Covered Number**	**Completeness (%)**	**Covered Number**	**Completeness (%)**	**Covered Number**	**Completeness (%)**
Golden	66	100%	55	98.21%	60	98.36%	63	96.92%
Red	65	98.48%	56	100%	60	98.36%	65	100%
Green	65	98.48%	56	100%	60	98.36%	65	100%
